# Photocurable Crosslinker from Bio-Based Non-Isocyanate Poly(hydroxyurethane) for Biocompatible Hydrogels

**DOI:** 10.3390/polym17091285

**Published:** 2025-05-07

**Authors:** Kathleen Hennig, Gabriele Vacun, Sibylle Thude, Wolfdietrich Meyer

**Affiliations:** 1 Department of Life Science and Bioprocesses, Fraunhofer Institute for Applied Polymer Research IAP, Geiselbergstr. 69, 14476 Potsdam-Golm, Germany; 2 Fraunhofer Institute for Interfacial Engineering and Biotechnology (IGB), Nobelstr. 12, 70569 Stuttgart, Germany; 3 Fraunhofer Institute for Manufacturing Engineering and Automation (IPA), Nobelstr. 12, 70569 Stuttgart, Germany; 4 Department of Functional Polymer Systems, Fraunhofer Institute for Applied Polymer Research IAP, Geiselbergstr. 69, 14476 Potsdam-Golm, Germany

**Keywords:** non-isocyanate polyurethane, photoreactive crosslinkers, cytocompatibility, green chemistry

## Abstract

This study explores the synthesis of photocurable non-isocyanate polyhydroxyethylurethanes (BPHUs) derived from renewable sources, designed for biomedical applications and the development towards advanced light curing processes. The following two pathways were developed: an aliphatic route using 1,4-butanediol-derived cyclic carbonates and an aromatic route with resorcinol-based carbonates. Ring-opening polymerization with dodecanediamine produced BPHU intermediates, which were methacrylated to form photoreactive derivatives (aliphatic MAs and aromatic MAs). Comprehensive characterization, including NMR, GPC, and FTIR, confirmed the successful synthesis. The UV curing of these methacrylated compounds yielded hydrogels with swelling properties. Aliphatic BPHUs achieved a gel content of 91.3% and a swelling of 1057%, demonstrating the flexibility and UV stability suitable for adaptable biomedical applications. Conversely, aromatic BPHUs displayed a gel content of 78.1% and a swelling of 3304%, indicating higher rigidity, which is advantageous for load-bearing uses. Cytotoxicity assessments adhering to the DIN EN ISO 10993-5 standard demonstrated non-cytotoxicity, with an >80% cell viability for both variants. This research underscores the potential of green chemistry in crafting biocompatible, versatile BPHUs, paving the way for eco-friendly materials in implantable medical devices.

## 1. Introduction: Green Synthesis from Bio-Based Source and Functionalization of Non-Isocyanate Poly(hydroxyurethane)

The drive toward sustainable materials has placed non-isocyanate polyurethanes (NIPUs) at the forefront of polymer research due to their eco-friendly synthesis and reduced toxicity. Unlike conventional polyurethanes, which rely on toxic isocyanates, one pathway for NIPU synthesis is via ring-opening reactions between cyclic carbonates and amines. This reaction produces polymers with primary and secondary hydroxyl groups (PHUs), imparting stability, environmental compatibility, and low toxicity. However, a key challenge is their typically low molecular weight, often resulting in oligomeric structures that limit their mechanical properties [1,2].

This limitation has been reframed as an advantage in the synthesis of photopolymerizable crosslinkers. Hydroxyethylurethane groups formed during the ring-opening process provide reactive sites for post-synthetic modification. Functionalization with acrylate photogroups in a second step enables these oligomeric BPHUs to be used as efficient UV-curable crosslinkers. Such crosslinkers exhibit rapid curing and the ability to achieve customizable crosslinking densities, thereby addressing the challenges in additive manufacturing. Jin et al. demonstrated how BPHU-based microspheres could achieve excellent photopolymer properties under UV light [3]. The same is true for the reactive diluents (not discussed in this work), which are the second main compound of each photoreactive resin and which was the focus of our earlier research, wherein we described with analogous non-isocyanate chemistry the monofunctional small molecules of the non-isocyanate urethanacrylates (NIUs), with the particle viscosities and high curability necessary for 3D bioprinting [4,5,6].

The potential of NIPUs extends to applications in advanced 3D printing technologies, particularly in the medical field. Modified NIPU crosslinkers have demonstrated their utility in creating photopolymerizable resins for medical implants, combining mechanical stability with biocompatibility. For instance, functionalized NIPUs were successfully applied to scaffold designs for tissue engineering, showcasing their adaptability in creating customized geometries with enhanced cell compatibility and biomimetic electrospuns functionalized with collagen [5,6]. Recent research has explored strategies to overcome the molecular weight limitations of NIPUs. For example, Oladzadabbasabadi et al. hybridized NIPUs with carboxymethyl cellulose, resulting in films with improved mechanical properties and functionality [7].

These findings suggest that the further optimization of synthesis conditions, coupled with functionalization strategies, can unlock new opportunities for NIPUs in photopolymer applications. The chemical synthesis of NIPUs, or, as discussed in this paper, polyhydroxyethans (PHEs), relies on cyclic carbonate precursors and amine-based curing agents to produce polyhydroxyurethane networks. These can be further modified post-synthetically to achieve desired mechanical and thermal properties [7]. In contrast, “green” photoreactive materials employ glycidyl azides and thiolene chemistry for sustainable photocrosslinking processes, yielding robust, biodegradable networks that are suitable for advanced applications, such as 3D printing [8,9].

Among the key components in the green synthesis of NIPUs are bis(2-oxo-1,3-dioxolan-4-yl)methylterephthalate and butanediol biscarbonate, both of which demonstrate the integration of bio-based and sustainable chemistry principles.

Bis(2-oxo-1,3-dioxolan-4-yl)methylterephthalate is an aromatic bis(cyclic carbonate) synthesized via the esterification of terephthalic acid, followed by cyclocarbonate formation. Derived from renewable feedstocks, such as biologically produced terephthalic acid, this precursor contributes to sustainable polymer chemistry. Its rigid aromatic structure enhances the thermal and mechanical stability of NIPUs, making it essential for applications like durable coatings and UV-curable systems [10,11]. Butanediol biscarbonate is produced through the cycloaddition of CO_2_ to epoxidized 1,4-butanediol, a reaction that effectively integrates CO_2_ as a renewable raw material. This process aligns with green chemistry principles, reducing greenhouse gas emissions while generating valuable cyclic carbonates. Innovations such as bifunctional organocatalysts and hybrid catalysts combining Beta-zeolites and ionic liquids have enhanced the efficiency and selectivity of this process under mild conditions [12,13,14]. These cyclic carbonates are pivotal for creating NIPUs, enabling environmentally beneficial applications such as solvent-resistant coatings and adhesives. Additionally, their utility extends to lithium-ion batteries, where they serve as patented components by advanced electrolytes, as in [15], thereby improving the performance and sustainability. Dodecandiamine, a key component in NIPU synthesis, has seen sustainable production advancements through biotechnological and green chemistry approaches. Engineered microorganisms like *Escherichia coli* and *Corynebacterium glutamicum* enable the production of diamines from renewable feedstocks, thus replacing fossil-based methods, as described by Wendisch et al. [16]. Similarly, enzymatic cascade catalysis converts cycloalkanes like cyclohexane into α,ω-diamines using biocatalytic one-pot systems, offering a high efficiency and sustainability [17]. Despite these advances, scaling Froidevaux accordingly remains a challenge, highlighting the need for the further optimization of the raw material availability and process efficiency [18]. But this work, dealing with bio-based polyhydroxyurethan (BPHU), certified the superior sustainability by utilizing renewable resources, enhanced hydrolytic stability, and versatile mechanical properties, presenting a viable alternative to conventional polyurethanes while addressing environmental concerns and enabling novel functionalization strategies, as Hanifa et al. states in [19]. Also, the production of the starting material of the introduced cyclic-carbonate educts, 1,4-butanediol (BDO), may be based on a renewable process utilizing sugarcane as the raw material, which is achieved through anaerobic fermentation with genetically engineered *Escherichia coli* strains [20] optimized for high yields and reduced by-product formation [21]. Synthetic biology approaches further enhance the metabolic pathways for sustainable production, addressing ecological and economic challenges [22] Similarly, 2,3-butanediol (2,3-BDO) can be produced using engineered *Saccharomyces cerevisiae* strains and wood residues as feedstock, offering significant environmental benefits over fossil-based methods [23,24]. In case of the production of the latter biscarbonate bis(2-oxo-1,3-dioxolan-4-yl)methylterephthalate, the plausible starting material would be bio-based terephthalic acid (TA), which can be synthesized using renewable resources through various innovative methods. The Diels–Alder reaction utilizes plant metabolites, sugars, or lignin to cycloadd acyclic 2,4-hexadiene (from fermentation), followed by dehydration and oxidation to bio-PTA [25]. The catalytic pyrolysis of lignocellulosic biomass, such as sawdust, produces p-xylene with a 23.4% yield, which is oxidized to TA with a 72.8% yield using metal oxide catalysts [26]. Finally, a solvent-free Diels–Alder reaction combines ethylene with 2,5-furandicarboxylic acid diethyl ester, catalyzed by Lewis acids, achieving up to a 59% yield with a high atom economy (Ogunjobi et al., 2019) [27]. These methods highlight the progress in sustainable TA production, but face challenges in commercial viability.

With this study, we aim to investigate the potential of bio-based materials synthesized through green chemical processes, focusing on their environmental and biomedical applications. Specifically, we address the following:

1. The development and investigation of bio-based polyhydroxyurethanes (BPHUs) as a specialized form of non-isocyanate polyurethanes (NIPUs), derived from NIPU chemistry. We focus on their synthesis routes, starting from bio-based cyclic carbonates and amines, exploring the effects of aromatic versus aliphatic precursors on the resulting materials.
2. The suitability of the synthesized BPHU compounds as alternative crosslinkers in hydrogels, specifically compared to the commonly used GelMa. These photocurable hydrogels are evaluated as HEAA photoinks for medical additive manufacturing applications, such as biocompatible implant structures.

## 2. Materials and Methods

### 2.1. Compounds

Bis[(2-oxo-1,3-dioxolan-4-yl)methyl] terephthalate and butandiol-Bis (cyclocarbonate) were purchased from specific polymers, France; DMF (reagent grade), THF (anhydrous), Methanol, N-hydroxyethylacrylamide (HEAA), Irgacure 184 (1-Hydroxycyclohexylphenylketo; 99%), and triethylamine (99%) were purchased from Sigma-Aldrich, Darmstadt Germany (now Merck, Darmstadt Germany). For the hydrogel preparation, two precursor solutions were formulated by dissolving the components in DMSO, targeting the following compositions (percentages likely by weight):

Aromatic Formulation: N-Hydroxyethylacrylamide (ca. 95%), aromatic methacrylated BPHU (product 4, ca. 5%), and Photoinitiator Irgacure 184 (0.5%).

Aliphatic Formulation: N-Hydroxyethylacrylamide (ca. 95%), aliphatic methacrylated BPHU (product 4′, ca. 5%), and Photoinitiator Irgacure 184 (0.5%).

The components were thoroughly mixed to ensure a homogeneous precursor solution. An appropriate volume of the precursor solution was then cast into molds (e.g., silicone molds or between glass plates with defined spacers) to obtain hydrogels with specific dimensions suitable for the intended tests (e.g., discs for swelling and biocompatibility).

Photopolymerization was initiated by exposing the cast precursor solution to UV-A light for 1 min using a high-intensity UV lamp (Dr. Hönle AG, UVAHAND 250, Gilching, Germany). After curing, the solid hydrogel samples were carefully removed from the molds. To remove the residual DMSO, photoinitiator fragments, and any unreacted monomers, first, this involved immersion in 70% aqueous ethanol, followed by extensive washing with an excess of distilled water over 24 h. The purified hydrogels were then used for further characterization.

Synthesis of BPHUs and Methacrylated Derivatives

**Figure 1:**
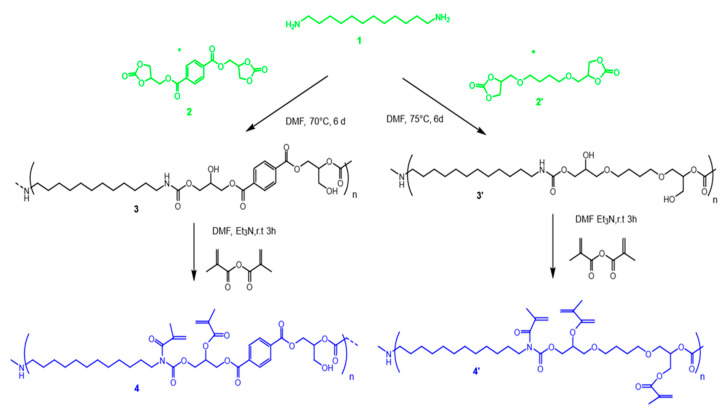
From biobased starting materials (green) and synthesis of the resulting polyhydroxyurethanes (BPHUs): one pathway involving an aromatic component (**left**) and the other following a purely aliphatic route(**right**). This was followed by a methacrylation step, resulting in the BPHU-MAs (blue).

Figure 1 illustrates the stepwise synthesis of the polyhydroxyurethanes (BPHUs) and their subsequent methacrylation.

In the left branch, aromatic dicyclic carbonate **2** reacts with diamine **1** in DMF at 70 °C over 6 days, forming the aromatic polyhydroxyurethane **3**. This intermediate **3** is further reacted with methacrylic anhydride in DMF and triethylamine (Et_3_N) at room temperature, introducing methacrylate functionalities and yielding the methacrylated derivatives, referred to as aromatic BPHU-MA (**4**) and aliphatic BPHU-MA (**4′**). In the right branch, aliphatic dicyclic carbonate **2′** undergoes a similar reaction with diamine 1 under slightly elevated conditions (DMF, 75 °C, 6 days), resulting in the aliphatic polyhydroxyurethane **3′**. Subsequent methacrylation of **3′** with methacrylic anhydride in the presence of Et_3_N produces the aliphatic methacrylated BPHU **4′**. This synthetic route demonstrates the modularity of BPHU formation and functionalization. The methacrylation step in particular introduces reactive double bonds, enabling further crosslinking or photopolymerization, which expands the application range of the BPHUs, particularly in advanced coatings, adhesives, or photopolymer systems.

### 2.2. Analytics

- Chromatography: The reactions were monitored by TLC (thin-layer chromatography) performed by using the POLYGRAM^®^ SIL G/UV254 from Macherey-Nagel, Düren, Germany. Column chromatography was performed by using Merck silica gel (particle size: 0.040–0.063 mm), Darmstadt, Germany.
- Gel Permeation Chromatography (GPC): The average molecular weights and molecular weight distributions (polydispersity index, PDI) of the produced polymers were determined by gel permeation chromatography (GPC). All of the measurements were performed on a Waters GPC system with a column of polysulfone styrenes. The GPC was equipped with a differential refractive index and UV detectors. Polymer solutions (2 mg/mL) were prepared in DMSO and filtered (1 μm PTFE), and then 100 μL of the solution was added to the column. The measurements were carried out at 80 °C with a flow rate of 1 mL/min. The samples were read by a UV detector at a wavelength of 280 nm. Pullulan standards in DMSO with 0.1 M LiBr were used for calibration.
- FTIR Spectroscopy: The Fourier transform infrared (FTIR) spectra were collected using an ATR-equipped Nicolet iS5 FTIR spectrometer (Thermo Fisher Scientific, USA). Samples of the NIPU intermediates and methacrylated derivatives (BPHU-MA) were recorded in the range of 4000–600 cm^−1^ with a resolution of 4 cm^−1^. Characteristic vibrations were monitored to confirm urethane formation (ν(N–H), ~1514 cm^−1^), the ring opening of cyclic carbonates (ν(C=O), 1809/1044 cm^−1^), and methacrylate functionalities (ν(C=C), 1640 cm^−1^).
- NMR spectra were measured on a Unity INOVA 500 NB spectrometer (Varian, Palo Alto, CA, USA) at 298 K. The ^1^H-NMR spectra and the ^13^C signals were recorded for the ^13^C-NMR spectra. Coupling constants (J) are reported in Hertz. Abbreviations to denote the multiplicity of a particular signal include s (singlet), d (doublet), t (triplet), dd (double doublet), q (quartet), and m (multiplet). The polymer composition was determined by ^1^H-NMR. The aromatic protons were set in relation to the hydroxy group of the HEAA component.
- Biocompatibility: The biocompatibility of methacrylated polyhydroxyurethanes (**BPHU 3** and **BPHU 4**) was evaluated following standardized cytotoxicity protocols. The materials were formulated into a hydrogel matrix and UV-cured for 1 min. Cytotoxicity testing was conducted by the accredited laboratory at Fraunhofer Stuttgart in accordance with DIN EN ISO 10993-5:2009 [28], which assesses the in vitro cytotoxic potential of medical devices. Extraction of the test material was performed following DIN EN ISO 10993-12:2012 [29] ensuring reproducible conditions for the preparation of the extracts. Human HaCaT keratinocyte cells were incubated with these extracts for 24 ± 2 h. Negative (fresh cell culture medium), positive (known cytotoxic substance), and blind controls (extraction medium subjected to the same extraction conditions as the material samples) were included in parallel to validate the test setup. After the incubation period, the cell viability was assessed to determine any cytotoxic effects. The results classified both materials as non-cytotoxic based on the criteria defined in the standard. This standardized approach ensured robust, reproducible, and internationally comparable results for assessing the cytocompatibility of hydrogel-based materials.
- Photostability: To assess the structural stability of the crosslinked hydrogels under prolonged irradiation, samples of aliphatic and aromatic BPHU-MA were exposed to continuous UV-A light (365 nm and 10 mW/cm^2^) for up to 42 h. At predefined time points (0 h, 2.5 h, 6 h, and 42 h), the samples were analyzed via ATR-FTIR spectroscopy using a Nicolet iS5 spectrometer (Thermo Fisher Scientific, USA) equipped with an iD7 ATR accessory. The IR spectra were collected in the range of 4000–600 cm^−1^ at a resolution of 4 cm^−1^ with 32 scans per measurement. Changes in the characteristic absorption bands, including ν(N–H), ν(OH), and ν(C=O), were evaluated to monitor potential degradation or structural changes in the polymer network.
- Determination of Swelling Degree: The swelling degree (*Q*) of the crosslinked polymer networks was determined to assess their ability to absorb aqueous fluids, an important parameter for biomedical applications. After determining the dry mass (*m_dry_*) of the polymer samples, they were immersed in distilled water at room temperature for a defined period (typically 24 h). Following incubation, the swollen samples were carefully removed, blotted to remove excess surface water, and immediately weighed (*m_swollen_*). The swelling degree was then calculated according to the following formula:
  $$Q=\frac{m_{swollen}-m_{dry}}{m_{dry}}\cdot 100\%$$
  where

*m_swollen_* = the mass of the swollen polymer after equilibrium swelling;

*m_dry_* = the initial dry mass of the polymer sample.

Water was chosen as the swelling medium due to its physiological relevance and the intended biomedical use of the polymeric coatings. The determined values reflect the hydrophilicity and network density of the polymer systems.

- Determination of Gel Content: To evaluate the efficiency of the crosslinking reaction in the photopolymerized networks, the gel content (G) was determined gravimetrically. Polymer films were first weighed in their dry state (initial mass: mpolymer), and then subjected to swelling in an appropriate solvent to remove all soluble, non-crosslinked fractions. After a defined swelling time, the remaining insoluble polymer was thoroughly dried and reweighed (mgew). The gel content was calculated using the following equation:
  $$G=\frac{m_{gew}}{m_{polymer}}\cdot 100\%$$
  where

*m_gew_* = the mass of the polymer after solvent extraction and drying;

*m_polymer_* = the initial dry mass of the polymer sample.

### 2.3. Synthesis

Poly[(N,N′-dodecane-1,12-diyl)bis(2-hydroxy-3-(1,3-dioxolane-4-yl)methylterephthalamide)] (arom**-BPHU**) **3** and Poly[(N,N′-dodecane-1,12-diyl)bis(2-(2-(meth)acryloyloxy)-3-(1,3-dioxolane-4-yl)methylterephthalamide)] (arom**-BPHU-MA 4**)

A solution of bis((2-oxo-1,3-dioxolan-4-yl)methyl) terephthalate (0.8637 g) and dodecandiamine (0.4725 g) in 6 mL of dry DMF was stirred at 70 °C under nitrogen atmosphere. After 6 days, the solution was cooled down to room temperature. The solution was diluted with 9 mL of dry DMF. A solution of methacrylic anhydride (0.1000 g) in DMF (1 mL) and triethylamine (0.09 mL) was added dropwise. After 3 h, the DMF was removed under reduced pressure. The residue was dissolved in Methanol and precipitated in diethyl ether. This procedure was repeated. The precipitate was dried in vacuo. Before (meth)acrylation, which yielded in 62%: ^1^H NMR (500 MHz, DMSO-d6) δ = 8.24–7.77 (4H), 7.36–6.67 (2H), 5.52–5.33 (1H), 5.10–5.02 (1H), 5.03–4.90 (1H), 4.41–4.14 (1H), 4.14–3.90 (1H), 3.70–3.50 (2H), 2.98–2.83 (4H), 1.43–1.26 (4H), and 1.26–0.80 (16H). After methacrylation: ^1^H NMR (500 MHz, DMSO-d6) δ = 8.24–7.77 (4H), 7.36–6.67 (1H), 6.06–5.93 (m, 0.14H), 5.61–5.53 (m,0.14H), 5.52–5.33 (1H), 5.32–5.24 (m, 0.14H) 5.10–5.02 (1H), 5.03–4.90 (1H), 4.41–4.14 (4H), 4.14–3.90 (1H), 3.70–3.50 (2H), 2.98–2.83 (4H), 1.85 (m, 0.42H), 1.43–1.26 (4H), and 1.26–0.80 (16H).

Poly[(N,N′-dodecane-1,12-diyl)bis(2-hydroxypropane-1,3-diyl-urethane)] **3′** and Poly[(N,N′-dodecane-1,12-diyl)bis(2-(2-(meth)acryloyloxy)propane-1,3-diyl-urethane)] **4′**

A solution of bis(cyclic carbonate) (0.8126 g) and dodecandiamine (0.5614 g) in 7 mL of dry DMF was stirred at 75 °C under nitrogen atmosphere. After 6 days, the solution was cooled down to room temperature. The solution was diluted with 9 mL of dry DMF. A solution of methacrylic anhydride (0.1000 g) in DMF (1 mL) and triethylamine (0.09 mL) was added dropwise. After 3 h, the DMF was removed under reduced pressure. The residue was dissolved in THF and precipitated in diethyl ether. This procedure was repeated. The precipitate was dried in vacuo. ^1^H-NMR (500 MHz, DMSO-d6) δ = 7.84 (br, 0.01H), 7.22–6.57 (2H), 6.01 (s, 0.01H), 5.66 (s, 0.01H), 5.61 (s, 0.02H), 5.27 (s, 0.02H), 5.08 (br, 0.02H), 4.98–4.82 (1H), 4.78–4.70 (1H), 4.70–4.63 (1H), 3.98–3.78 (2H), 3.78–3.66 (1H), 3.51–3.41 (4H), 3.41–3.34 (4H), 3.34–3.22 (2H), 3.01–2.87 (4H), 1.86 (s, 0.03H), 1.83 (s, 0.9H), 1.58–1.43 (4H), 1.43–1.29 (4H), and 1.29–1.07 (16H).

## 3. Results

### 3.1. FTIR Analysis of BPHU Formation with Aromatic and Aliphatic Dicyclic Carbonates

**Figure 2:**
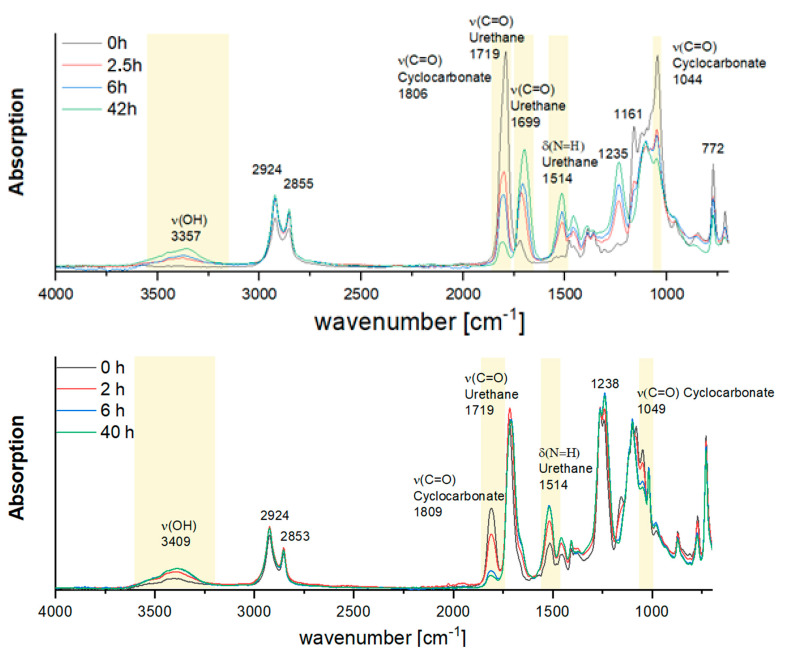
FTIR spectra before and after the reaction for the synthesis of polyhydroxyurethanes (**BPHUs 3** and **4**) from aromatic (**top panel**) and aliphatic (**bottom panel**) dicyclic carbonates. Key bands: carbonate (1044/1806 cm^−1^), urethane (1699/1719 cm^−1^), and N–H (1514 cm^−1^).

The FTIR analysis in Figure 2 demonstrates the successful formation of polyhydroxyurethanes (BPHUs) through the reactions of amines with both aromatic and aliphatic dicyclic carbonates (**2** and **2′**, respectively). For the aromatic BPHU **3** synthesis (upper spectra in Figure 2), the characteristic C=O stretching vibrations of the cyclic carbonate at 1806 cm^−1^ and 1044 cm^−1^ decrease over time, indicating the consumption of carbonates. Concurrently, the C=O urethane band at 1699 cm^−1^ and the N–H deformation peak at 1514 cm^−1^ intensify, confirming the formation of urethane linkages. The increase in the broad O–H stretching vibration at 3357 cm^−1^ reflects the generation of hydroxyl groups during the reaction. For the aliphatic BPHU **3′** synthesis (lower spectra in Figure 2), a similar trend is observed. The C=O carbonate vibrations at 1809 cm^−1^ and 1049 cm^−1^ diminish, while the urethane-specific C=O band at 1719 cm^−1^ and the N–H deformation peak at 1514 cm^−1^ grow over time. The increase in the O–H band at 3409 cm^−1^ further supports the formation of hydroxyl-functional polyurethanes. The shift difference in the carbonate and urethane peaks between the aromatic and aliphatic derivatives is attributed to the differing rigidity and electron density of the backbones, which influences hydrogen bonding and dipole interactions in the formed urethane linkages.

These results align well with analogous chemistries involving dicyclic carbonates and showcase the efficient, isocyanate-free synthesis of BPHUs with aromatic and aliphatic backbones under mild conditions.

### 3.2. GPC Analysis of Methacrylated BPHUs

The GPC results for the methacrylated BPHUs reveal significant differences between the aromatic and aliphatic variants, particularly in terms of the molecular weight, polydispersity index (PDI), and yields (see Table 1). As only a single measurement was performed, an estimated method uncertainty of ±5% is assumed for the molecular weight and PDI values.

The aromatic BPHU demonstrates a high weight-average molecular weight (M_w_) of 42.974 g/mol, with a significantly lower number-average molecular weight (M_n_) of 2631 g/mol, resulting in a broad PDI of 17.4. This broad distribution reflects the substantial heterogeneity in the chain length, possibly due to incomplete polymerization or reactivity differences during the synthesis. The relatively low yield of 62.2% further supports the possibility of side reactions or incomplete monomer conversion.

In contrast, the aliphatic BPHU exhibits a much lower M_w_ of 2867 g/mol, with an M_n_ of 1905 g/mol, leading to a narrow PDI of 1.5. This narrower distribution indicates a more uniform chain length and likely reflects a more controlled polymerization process. The significantly higher yield of 86.6% suggests more efficient polymer formation, likely due to fewer side reactions or better monomer compatibility. The GPC results highlight distinct differences in the polymerization behavior between the aromatic and aliphatic BPHUs. The aromatic BPHU’s broad molecular weight distribution and lower yield suggest challenges in achieving consistent material properties and efficient monomer utilization. This could affect the scalability and reproducibility in industrial applications. On the other hand, the aliphatic BPHU’s narrow molecular weight distribution and higher yield indicate a more efficient and uniform polymerization process, which could lead to more consistent material performance during processing and application.

### 3.3. Biocompatibility Testing

**Figure 3:**
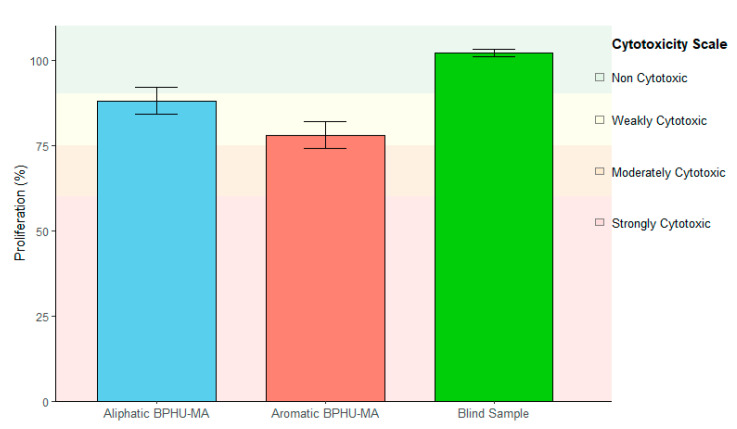
Cytotoxicity of the 5% crosslinkers **4** and **4′** in a HEAA hydrogel.

The biocompatibility evaluation of the methacrylated polyhydroxyurethane (BPHU-MA) hydrogels revealed promising results for both the aliphatic and aromatic derivatives as shown in Figure 3. Using a hydrogel composition with a 5% crosslinker based on BPHU-MA, the materials demonstrated cell viability values of 87.8% ± 3.5% (relative to the negative control) for the aliphatic BPHU-MA, resulting in a non-cytotoxic potential. The aromatic BPHU-MA showed a cell viability of 78.0% ± 4.0% (relative to the negative control). This value is at the threshold of mild cytotoxicity but, according to the less stringent ISO standards, it can still be considered non-cytotoxic. The blind control showed a proliferation rate of 102.4%, confirming that the extraction containers are non-cytotoxic, and any cytotoxic effect can be clearly attributed to the materials tested. Furthermore, a positive control was used to show that the cells react to a cytotoxic substance. The reliability and validity of the test method were thus demonstrated.

### 3.4. Gel Content and Swelling Behavior

Hydrogels synthesized from the crosslinked aliphatic BPHU-MA exhibited a gel content of 91.3 ± 2.9%, while those based on the crosslinked aromatic BPHU-MA showed a lower value of 78.1 ± 1.6%, indicating a higher crosslinking density in the aliphatic system. The swelling behavior displayed an inverse trend: the aliphatic BPHU-MA hydrogels absorbed water moderately (1057 ± 98%), whereas the aromatic BPHU-MA hydrogels showed a significantly higher swelling ratio (3304 ± 180%). This enhanced water uptake in the aromatic variant is attributed to its lower crosslinking density and greater free volume within the network. These observations suggest that the crosslinked aromatic BPHU-MA forms looser, more hydrophilic structures, while the aliphatic BPHU-MA yields more compact and denser hydrogel networks due to its flexible backbone.

### 3.5. UV Stability

To evaluate the photochemical stability of the hydrogels, the samples of crosslinked aliphatic and aromatic BPHU-MA were subjected to continuous UV-A irradiation (365 nm and 10 mW/cm^2^) over a period of 42 h. Structural changes were monitored using ATR-FTIR spectroscopy (Thermo Fisher Nicolet iS5, equipped with an iD7 ATR module) at distinct time intervals (0 h, 2.5 h, 6 h, and 42 h). Key absorption bands corresponding to urethane linkages (ν(N–H) ≈ 1514 cm^−1^), hydroxyl functionalities (ν(OH) ≈ 3350–3400 cm^−1^), and carbonyl groups from residual cyclic carbonates (ν(C=O) ≈ 1806–1809 cm^−1^) remained consistent in both systems throughout the irradiation period. These results indicate the high structural integrity of the polymer networks under UV exposure. Slightly better signal retention was observed in the aliphatic BPHU-MA, suggesting enhanced photostability compared to the aromatic counterpart.

### 3.6. ^1^H- and ^13^C-NMR Analysis and Degree of Methacrylation (DoM) of Endproducts

Proton Nuclear Magnetic Resonance (^1^H-NMR) spectroscopy was employed not only to confirm the successful methacrylation of the BPHU intermediates and verify the structures of the final photoreactive endproducts, aromatic BPHU-MA (**4**), and aliphatic BPHU-MA (**4′**), but also to quantify the extent of the functionalization. The obtained spectra display characteristic signals corresponding to the methacrylate groups (e.g., vinyl protons ~5.6–6.1 ppm and methyl protons ~1.85 ppm) alongside the BPHU backbone protons.

Based on the integration of these signals, specifically comparing the methacrylate methyl protons to the internal methylene protons of the dodecanediamine backbone as an internal reference, the Degree of Methacrylation (DoM) was calculated. This analysis yielded DoM values of 3.5% for the aromatic BPHU-MA (**4**) and 7.75% for the aliphatic BPHU-MA (**4′**). Detailed ^1^H-NMR spectra, peak assignments, the methodology for the DoM calculation, and further discussion on its implications are provided in the Supplementary Materials.

## 4. Conclusions

This study successfully demonstrated the synthesis and characterization of bio-based non-isocyanate poly(hydroxyurethane) (BPHU) crosslinkers for photocurable hydrogel applications. The developed synthetic pathways, utilizing both aliphatic and aromatic cyclic carbonate precursors, highlight the potential of green chemistry in designing sustainable polymeric materials.

Key findings from our work include the efficient conversion of dicyclic carbonates into photoreactive BPHU intermediates, as confirmed by the FTIR, NMR, and GPC analyses. The subsequent methacrylation of these intermediates yielded UV-curable hydrogels with distinct properties tailored to specific biomedical applications. The aliphatic BPHU-based hydrogels exhibited a higher flexibility, UV stability, and a swelling ratio of 1057%, making them suitable for adaptable biomedical applications. In contrast, the aromatic BPHU-based hydrogels demonstrated higher rigidity with a swelling ratio of 3304%, potentially benefiting load-bearing biomedical applications.

Furthermore, the cytotoxicity assessments according to the DIN EN ISO 10993-5 standard confirmed the biocompatibility of both hydrogel variants, with cell viability exceeding 80%. These results validate the potential of BPHU-based materials as safe and effective alternatives to conventional polyurethanes in medical applications, particularly for potential development in advanced 3D printing and tissue engineering.

Overall, this study underscores the viability of BPHUs as environmentally friendly and biocompatible alternatives for photocurable crosslinkers. Although direct mechanical data were not recorded in this study, the high swelling ratio observed for aromatic BPHU-MA (3304%) implies a denser, stiffer network. Future work will include comparative mechanical characterization to benchmark against commercial hydrogels such as PEGDA or GelMa systems. Further development of these novel materials for medical additive manufacturing could pave the way for sustainable, high-performance biomaterials with significant clinical and environmental benefits.

Future research will aim at incorporating bioactive motifs and evaluating the materials’ performance in vivo, especially in applications such as tissue scaffolds or wound dressings.

## Figures and Tables

**Table 1:** GPC and yields of the synthesized methacylated BPHU-MA.

	Aromatic BPHU-MA 4	Aliphatic BPHU-MA 4′
M_w_ [g/mol]	42.974	2867
M_n_ [g/mol]	2631	1905
PDI	17.4	1.5
Yield [%]	62	86

## Data Availability

All data are presented in the manuscript.

## Supplementary Materials

The following supporting information can be downloaded at: https://www.mdpi.com/article/10.3390/polym17091285/s1. Figure S1: Aromatic BPHU-MA (compound **4** in the main text); Figure S2: ^1^H NMR spectrum (500 MHz, DMSO-d6) of aromatic BPHU-MA (1-MA, compound 4); Figure S3: ^13^C NMR spectrum (126 MHz, DMSO-d6) of aromatic BPHU-MA (1-MA, compound 4); Figure S4: Aliphatic BPHU-MA (compound 4′ in main text); Figure S5: ^1^H NMR spectrum (500 MHz, DMSO-d6) of aliphatic BPHU-MA (4′). Inlet zoom of small siganls); Figure S6: ^13^C NMR spectrum (126 MHz, DMSO-d6) of aliphatic BPHU. Precourser of methacryleted aliphatic BPHU-MA 4. The supplementary material describes the use of ^1^H and ^13^C NMR spectroscopy to confirm the successful synthesis and methacrylation of biobased poly(hydroxyurethane)s (BPHU-MA). Spectral data support the structural assignment of key functional groups, including urethane, hydroxyl, and methacrylate moieties. The degree of methacrylation (DoM)was estimated from integrated NMR signals, and its relevance for photocuring and hydrogel formation is discussed.
