# Supplementary material for: Photocurable Crosslinker from Bio-Based Non-Isocyanate Poly(hydroxyurethane) for Biocompatible Hydrogels
Source: Polymers (Basel). 2025 May 7;17(9):1285. doi: 10.3390/polym17091285 (PMC12073706; doi:10.3390/polym17091285)
Supplement: Supplementary file 1 [file polymers-17-01285-s001.zip › polymers-3578193-supplementary.pdf]

Supporting Information

Photocurable Crosslinker from Biobased Non-Isocyanate Poly(hydroxyurethane)s for Biocompatible Hydrogels

Kathleen Hennig [1,*], Gabriele Vacun [2], Sibylle Thude [3] and Wolfdietrich Meyer [1,4,*]

[1] Department of Life Science and Bioprocesses, Fraunhofer Institute for Applied Polymer Research IAP, Germany
[2] Fraunhofer Institute for Interfacial Engineering and Biotechnology (IGB), Stuttgart, Germany
[3] Fraunhofer Institute for Manufacturing Engineering and Automation (IPA), Stuttgart, Germany
[4] Department of Functional Polymer Systems, Fraunhofer Institute for Applied Polymer Research IAP, Germany
* Correspondence: wolfdietrich.meyer@iap.fraunhofer.de, kathleen.hennig@iap.fraunhofer.de ; Tel.: +49 331 568 1442,

NMR Spectroscopy

Nuclear Magnetic Resonance (NMR) spectroscopy, specifically ^{1}H and ^{13}C NMR, was utilized to confirm the successful synthesis of the methacrylatd BPHUs. Spectra were recorded in DMSO-d6.
The experimental 13C NMR chemical shifts were assigned by comparison with the predicted values generated using ChemDraw Professional (version 23.11, PerkinElmer). The predicted chemical shifts (blue numbers in chemical structures as inlets) in the facilitated the unambiguous assignment of the observed resonances to the corresponding carbon atoms in the molecular structure.

Characterization of Aromatic BPHU-MA

The ^{1}H NMR spectrum of the aromatic BPHU-MA (Figure 1) is shown in Figure 2Figure 2. The spectrum displays signals consistent with the desired structure, including the aromatic terephthalate unit, the dodecanediamine backbone, the urethane and hydroxyl functionalities, and the introduced methacrylate groups:

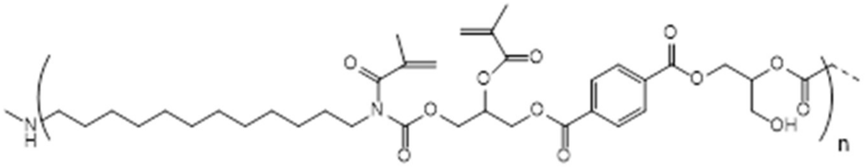

Figure S1: aromatic BPHU-MA (compound 4 in the main text)

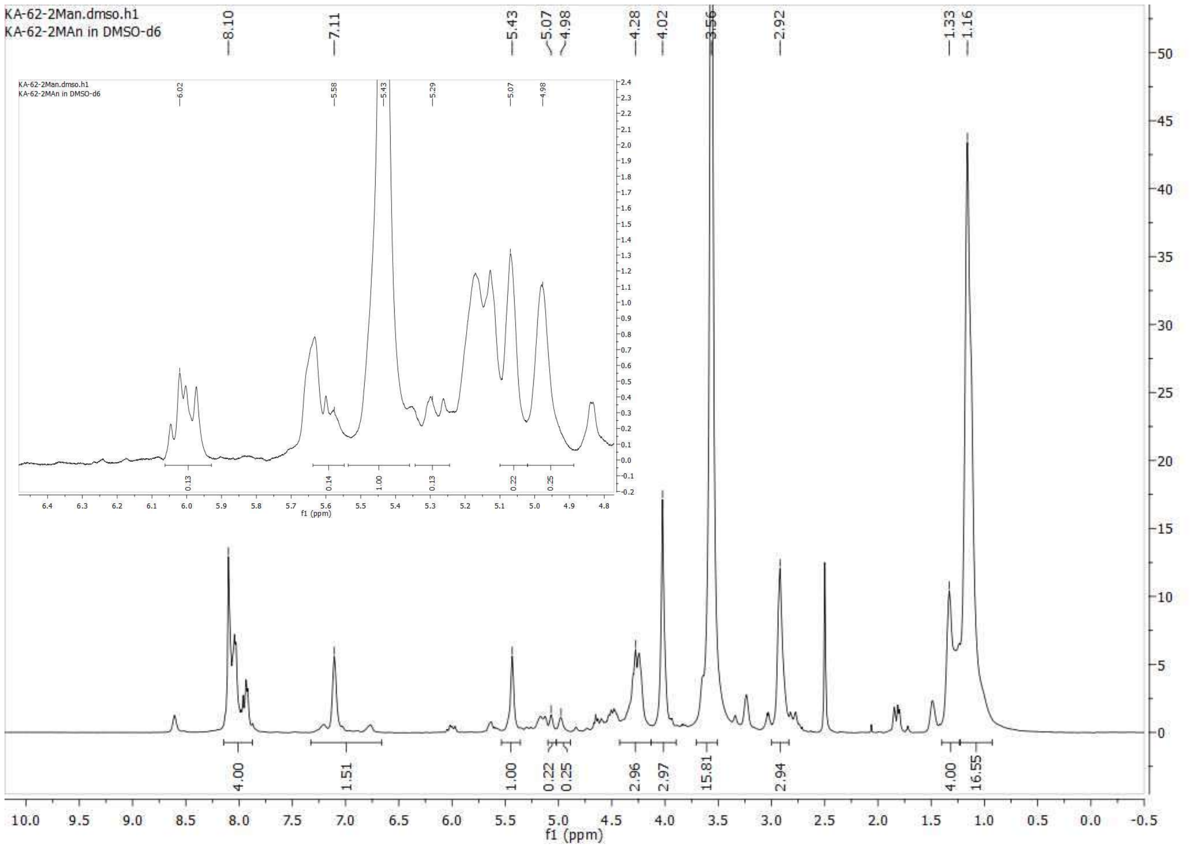

Figure S2:Figure S.X1. ^{1}H NMR spectrum (500 MHz, DMSO-d6) of aromatic BPHU-MA (1-MA, compound 4).

- δ 8.24 – 7.77 ppm (4H): Protons of the terephthalate aromatic ring.
- δ 7.36 – 6.67 ppm (2H, broad): Urethane N-H protons.
- δ 5.52 – 5.33 ppm (1H), 5.10 – 5.02 ppm (1H), 5.03 – 4.90 ppm (1H), 4.41 – 4.14 ppm (1H), 4.14 – 3.90 ppm (1H), 3.70 – 3.50 ppm (2H): Protons in the hydroxyurethane linkages.
- δ 2.98 – 2.83 ppm (4H): Methylene protons adjacent to nitrogen in dodecanediamine.

- δ 1.43 – 1.26 ppm (4H): Methylene protons distal to nitrogen in dodecanediamine.
- δ 1.26 – 0.80 ppm (16H): Internal methylene protons of dodecanediamine.
- δ 6.06 – 5.93 ppm (m, 0.14H), 5.61 – 5.53 ppm (m, 0.14H), 5.32–5.24 ppm (m, 0.14H): Methacrylate vinyl protons.
- δ 1.85 ppm (m, 0.42H): Methacrylate methyl protons.

The ^{13}C NMR spectrum of aromatic BPHU-MA (1-MA) is presented Figure 3. Key signals include:

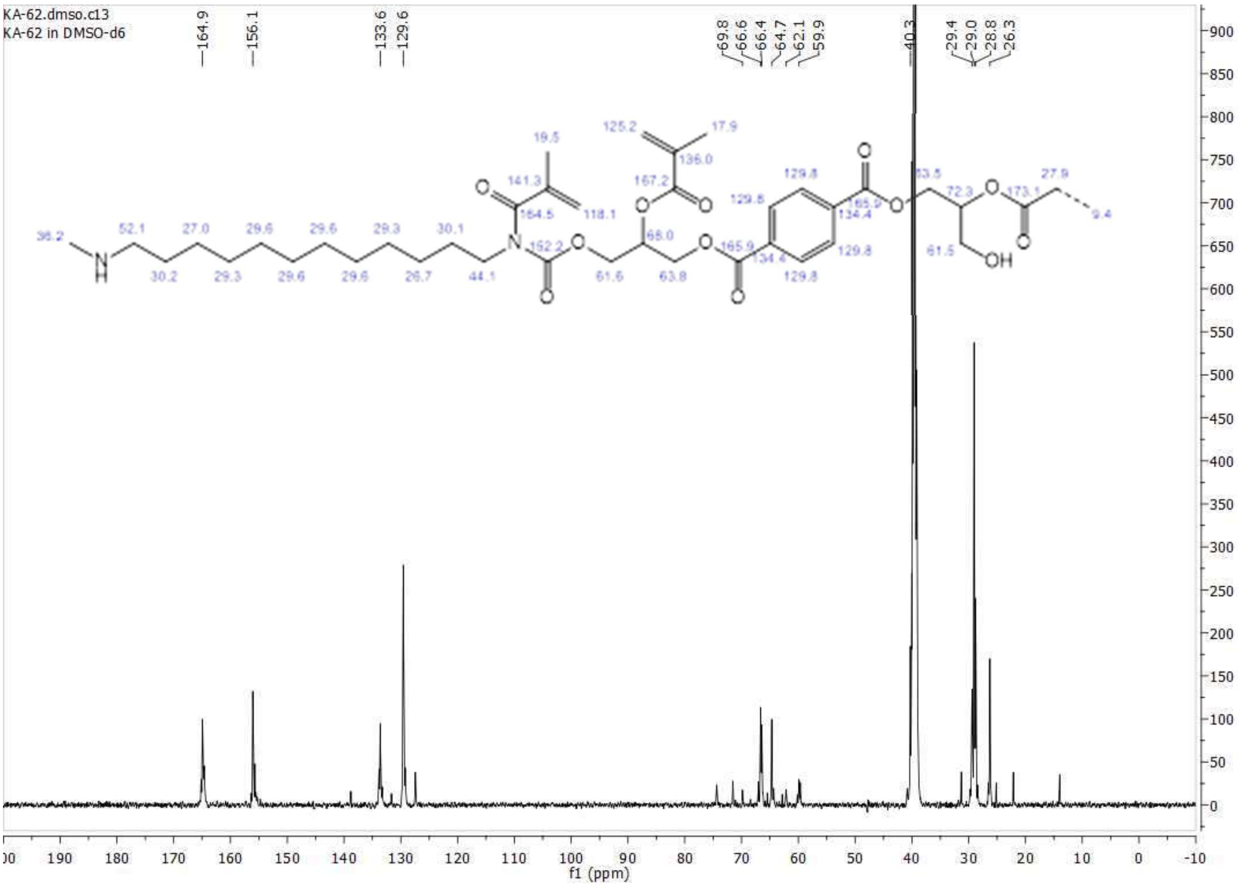

Figure S3: ^{13}C NMR spectrum (126 MHz, DMSO-d6) of aromatic BPHU-MA (1-MA, compound 4).

- δ 126-130 ppm and around 164 ppm: Aromatic ring carbons.
- δ 156.1 ppm: Urethane carbonyl carbon (C=O).
- δ 164.9 ppm: Methacrylate ester carbonyl carbon (C=O).
- δ 133.6 ppm and 129.6 ppm: Methacrylate vinyl carbons (C=C).
- δ 26-70 ppm: Dodecanediamine and hydroxyurethane backbone carbons.

The ¹H NMR spectrum of the aliphatic BPHU-MA (Figure 4) in Figure 5 confirms the successful synthesis and methacrylation of the aliphatic BPHU structure:

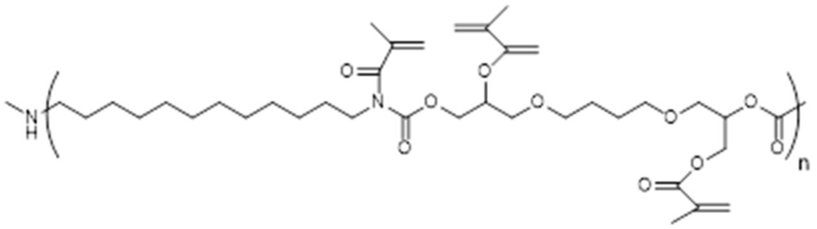

Figure S4:aliphatic BPHU-MA (compound 4' in main text)

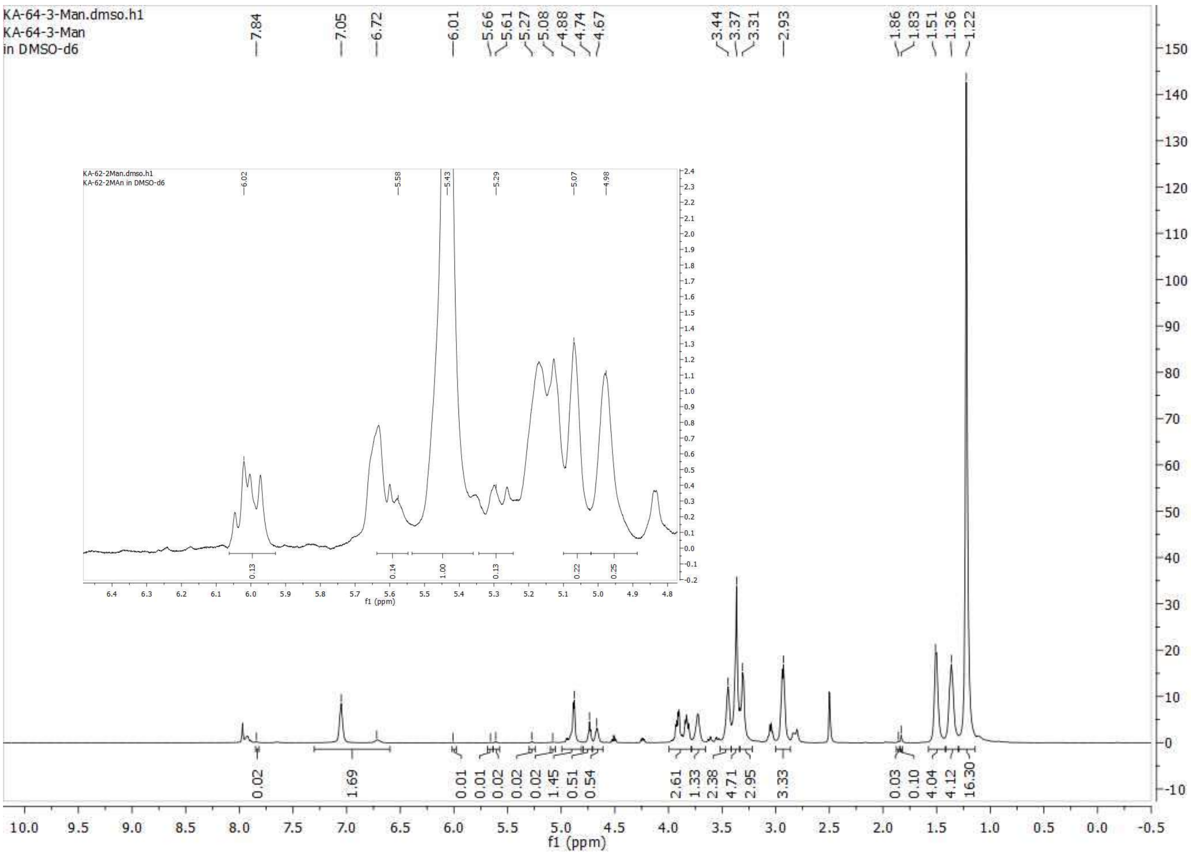

*Figure S5: ¹H NMR spectrum (500 MHz, DMSO-d6) of aliphatic BPHU-MA (**4'**). Inlet zoom of small siganls).*

- δ 7.84 ppm (br, 0.01H): Urethane N-H protons (*Note: Integration appears very low*).
- δ 4.98 – 4.82 ppm (1H), 4.78 – 4.70 ppm (1H), 4.70 – 4.63 ppm (1H), 3.98 – 3.78 ppm (2H), 3.78 – 3.66 ppm (1H), 3.51 – 3.41 ppm (4H), 3.41 – 3.34 ppm (4H), 3.34 – 3.22 ppm (2H): Protons in the hydroxyurethane linkages and aliphatic backbone.
- δ 3.01 – 2.87 ppm (4H): Methylene protons adjacent to nitrogen in dodecanediamine.
- δ 1.58 – 1.43 ppm (4H), 1.43 – 1.29 ppm (4H), 1.29 – 1.07 ppm (16H): Internal methylene protons of dodecanediamine.
- δ 6.01 ppm (s, 0.01H), 5.66 ppm (s, 0.01H), 5.61 ppm (s, 0.02H): Methacrylate vinyl protons.
- δ 1.86 ppm (s, 0.03H), 1.83 ppm (s, 0.9H): Methacrylate methyl protons.

The ^{13}C NMR spectrum of aliphatic BPHU shows characteristic signals:

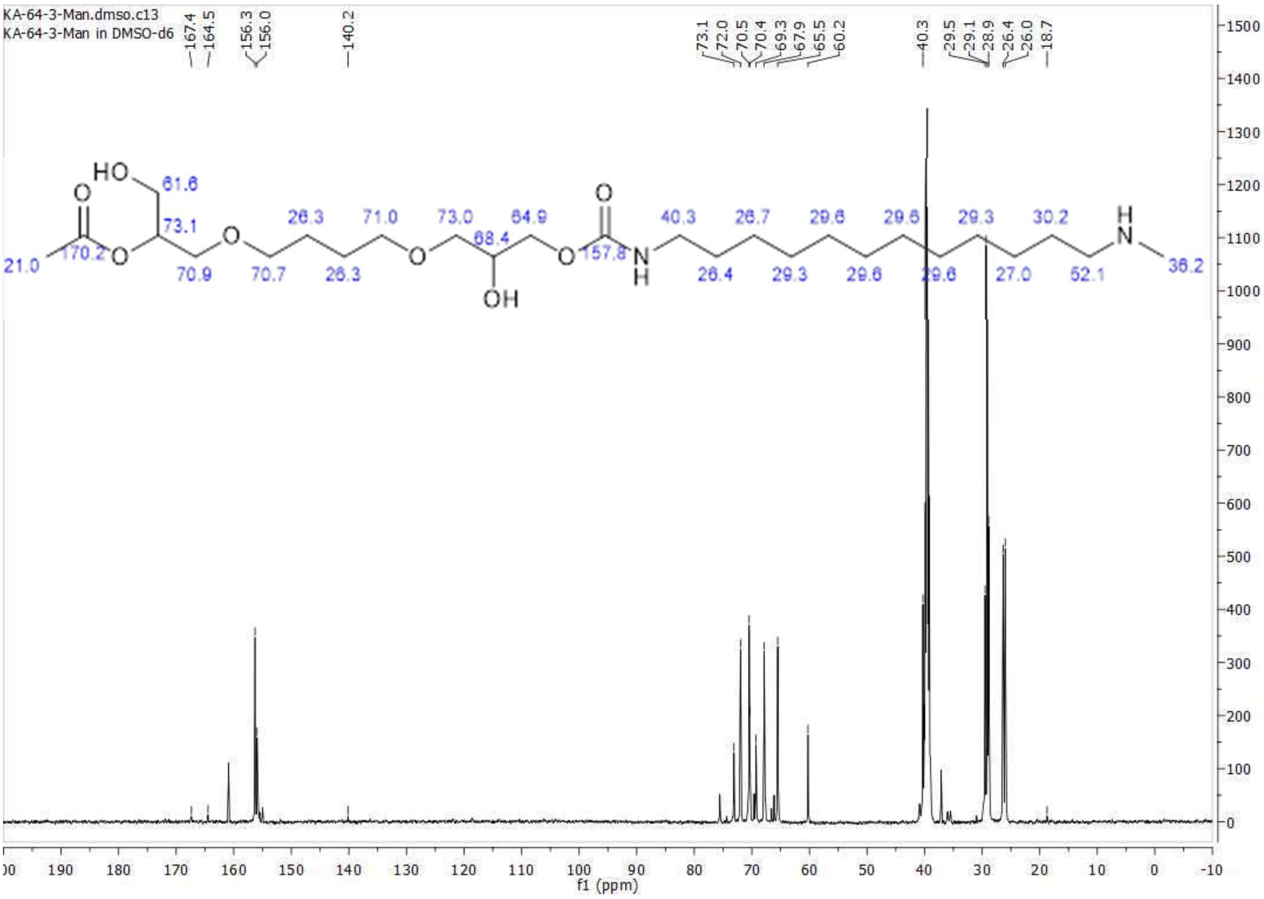

Figure S6: ^{13}C NMR spectrum (126 MHz, DMSO-d6) of aliphatic BPHU. Precourser of methacryleted aliphatic BPHU-MA 4.

- δ 167.4 ppm and 164.5 ppm, δ 156.3 ppm and 156.0 ppm: Urethane carbonyl carbons (C=O, two signals indicating non-equivalence).
- δ 18.7-73.1 ppm: Dodecanediamine and hydroxy urethane backbone carbons.

Determination of Degree of Methacrylation (DoM)

The degree of methacrylation (DoM) quantifies the average number of methacrylate groups attached per polymer repeat unit and is crucial for predicting crosslinking density. Each repeat unit in our BPHU structures, formed by the reaction of a bis-cyclic carbonate with dodecanediamine, contains four potential hydroxyl groups (two primary and two secondary) available for methacrylation.

The DoM was estimated from the ^{1}H NMR spectra by comparing the integral of the methacrylate methyl protons (δ ~1.8 ppm, corresponding to 3 protons per methacrylate group) to the integral of a specific, well-defined signal from the polymer backbone corresponding to a known number of protons per repeat unit. The internal methylene protons of the dodecanediamine segment (δ 1.26 – 0.80 ppm for 1-MA, δ 1.29 – 1.07 ppm for 2-MA), which integrate to 16 protons per repeat unit, were used as the internal standard.

The DoM was calculated using the following formula:

Aromatic BPHU-MA

DoM = (0.42 ÷ 3 ÷ (16 ÷ 16)) × (1 ÷ 4) × 100% = **3.5%**

Aliphatic BPHU-MA (2-MA):

DoM = (0.93 ÷ 3 ÷ (16 ÷ 16)) × (1 ÷ 4) × 100% = **7.75%**

Applying this formula using the integrated peak areas listed in the experimental section of the main paper:

- Aromatic BPHU-MA (1-MA):
  - Integral Methacrylate Methyl (δ 1.85 ppm): 0.42H
  - Integral Backbone Reference (δ 1.26-0.80 ppm): 16H

- Aliphatic BPHU-MA (2-MA):
  - Integral Methacrylate Methyl (sum of signals at δ 1.83 and 1.86 ppm): 0.03H + 0.9H = 0.93H
  - Integral Backbone Reference (δ 1.29-1.07 ppm): 16H

Limitations and Interpretation of DoM:

The calculated DoM values based on the provided ^{1}H NMR integrations appear notably low, suggesting that only a small fraction of the available hydroxyl groups were functionalized under the reaction conditions. These values also lead to inconsistencies when comparing the calculated number of methacrylate groups (0.14 for 1-MA, 0.31 for 2-MA) to the integrations of the methacrylate vinyl protons listed in the same dataset (e.g., total vinyl integration for 2-MA is listed as 0.04H, while 0.31 groups would correspond to 0.31 * 2 = 0.62H). These

discrepancies in integration values make a precise, quantitative determination of the DoM from this dataset challenging.

Despite the apparent low DoM values derived from these specific integrations, both materials were successfully photo-crosslinked into solid hydrogels, as evidenced by the gel content analysis (main paper, Section 3.4). This indicates that a sufficient density of photoreactive methacrylate groups was present to form a covalent network upon UV irradiation. The observed photopolymerization ability and the resulting hydrogel properties (swelling behavior, gel content) are functional indicators of the materials' suitability as crosslinkers. While further optimization of reaction conditions or alternative quantitative methods (e.g., titration) could be employed for a more accurate DoM determination, the presented NMR data clearly confirm the presence of the methacrylate functionality on the synthesized BPHU backbone, validating their potential as photocurable crosslinkers for additive manufacturing.
